# Effects of trabeculectomy on the postoperative central visual field as revealed by refraction values

**DOI:** 10.1007/s10384-024-01139-2

**Published:** 2024-11-08

**Authors:** Kosuke Nakajima, Rei Sakata, Shiroaki Shirato, Makoto Aihara

**Affiliations:** 1 Department of Ophthalmology, The University of Tokyo Hospital, 7-3-1 Hongo, Bunkyo-ku, Tokyo, 113-8655 Japan; 2 Department of Ophthalmology, The University of Kyorin Hospital, Tokyo, Japan; 3 Yotsuya Shirato Eye Clinic, Tokyo, Japan

**Keywords:** Normal-tension glaucoma, Refractive myopia, Central visual field, Trabeculectomy

## Abstract

**Purpose:**

To evaluate the effects of trabeculectomy on the rate of deterioration of the central visual field (VF) in patients with normal-tension glaucoma (NTG), as revealed by refraction values.

**Study design:**

Retrospective case series.

**Methods:**

We retrospectively analyzed 28 eyes, including 12 high myopic (spherical equivalent [SE] < ˗ 6 diopters without pathological myopia) and 16 non-high myopic (SE ≥ – 6 diopters) eyes. The rate of VF deterioration (dB/year) was determined using linear regression analysis of 30 -2 and 10 -2 VF tests. The Wilcoxon signed-rank test was used to compare deterioration rates between groups. To assess the influence of initial post-surgery effects, statistical analyses were conducted with and without data from the initial postoperative VF exam.

**Results:**

Trabeculectomy significantly reduced intraocular pressure (IOP) in myopic (14.1 to 9.0 mmHg, *P* ≤ 0.01) and non-myopic (13.4 to 9.5 mmHg, *P* ≤ 0.01) eyes. Postoperatively, the 10-2 VF deterioration rate significantly decreased in myopic (˗ 1.31 to ˗ 0.55 dB/year, *P* = 0.01) and non-myopic (– 0.80 to – 0.30 dB/year, *P* = 0.03) eyes. Excluding the first postoperative VF exam, the deterioration rates were – 0.51 ± 0.24 dB/year and – 0.54 ± 0.89 dB/year, respectively, indicating a minor impact on progression assessment.

**Conclusions:**

Trabeculectomy may mitigate central VF deterioration in myopic NTG patients, emphasizing the potential benefits of timely surgical intervention. Further studies are needed to determine the optimal timing for surgery.

## Introduction

Myopia is a very common condition worldwide [1, 2]. The proportion of patients with both myopia and glaucoma is estimated to be relatively high [3]. The papillary macular fiber bundles of the disc are more likely to be affected in myopic eyes with normal-tension glaucoma (NTG); this often affects the central visual field (VF) and compromises visual function [4]. The central VF is particularly important for maintenance of visual function and prevention of blindness in patients with glaucoma; the central VF function and quality of life are closely correlated [5]. The impact of myopia on NTG progression remains unclear [6–8]; some reports suggest that a lower degree of myopia protects against glaucoma progression [9–12]. Although several studies report that trabeculectomy with mitomycin C (MMC) administration effectively treats NTG [13–17], refractive values have not been used to study the efficacy of such treatment in glaucoma eyes with NTG. One study on filtration and implant surgery to treat myopic primary open-angle glaucoma (POAG) found that eyes with a larger β-peripapillary atrophic area (PPA) exhibited faster progression of central VF defects after surgery and a more rapid rate of VF deterioration than non-myopic eyes [18]. However, the surgical techniques differed; moreover, the preoperative VF deterioration rate was not discussed.

When comparing the rate of deterioration before and after filtering surgery, it is important to consider whether the deterioration persists postoperatively; some reports describe further worsening of VF function after trabeculectomy [19, 20]. Postoperative progression of VF might thus differ according to whether the VF data from the first postoperative examination are included in the analysis. However, there is a need for reliable long-term data to confirm this.

In our previous report [21], we used long-term data to examine the effects of trabeculectomy on various components of the VF. In the present exploratory study, we investigated the impact of trabeculectomy on the central VF based on the refractive values. Considering that the rate of preoperative progression may not have slowed for the first 6 months postoperatively, we evaluated progression both when data from the first postoperative VF test were included and when they were omitted.

## Materials and methods

In this retrospective cohort study, we reviewed the medical records of Japanese patients with NTG who had undergone maximally tolerated medical treatments followed by initial fornix-based trabeculectomy with MMC between January 2011 and December 2013. The study was conducted in accordance with the principles of the Declaration of Helsinki. The research was retrospective, being based on data collected for clinical purposes. Ethical approval was granted by the Institutional Review Board of the Riverside Internal Medicine Cardiology Clinic (approval ID: RSC-1811RB01). Written informed consent was obtained from all patients.

The inclusion criteria were age < 80 years; diagnosis of NTG was using the standardized criteria specified below; follow-up for ≥ 5 years before and after surgery each; fornix-based trabeculectomy performed by a single well-trained surgeon (SS) at Yotsuya Shirato Eye Clinic (Tokyo, Japan); a single or combined procedure with phacoemulsification procedure; and VF tests conducted every 6 months using a Humphrey Visual Field Analyzer (Carl Zeiss Meditec) running the Swedish Interactive Threshold Algorithm (SITA) standard 30-2 and 10-2 programs, with the following VF test reliability indices: fixation < 20%, false-positive rate < 15%, and false-negative rate < 15%. We included eyes with consistently reliable VF tests. We included patients who underwent reliable 10-2 VF tests on ≥ 6 occasions both before and after surgery. Preoperative observation revealed a pattern of progressive VF deterioration, characterized by the enlargement of existing defects and the development of new defects in previously healthy VF regions [22]. The exclusion criteria were intraocular lenses or nuclear cataracts before and after surgery; macular changes or degeneration on optic coherence tomography (OCT) images; and previous refractive corneal surgery.

A total of 352 patients underwent trabeculectomy during the study period, with or without combined cataract surgery. Among these eyes, 56 met the above inclusion and exclusion criteria. Of these eyes, only 28 (of 28 patients) yielded consistently reliable VF test results throughout the entire observation period [21].

NTG was diagnosed according to the Japan Glaucoma Society guidelines (5th edition) [23]. The diagnostic criteria included [24] characteristic changes in the optic nerve head with corresponding VF defects as determined using the Humphrey Visual Field Analyzer; intraocular pressure (IOP) (measured using a Goldmann applanation tonometer; Haag-Streit) that never exceeded 21 mmHg without any ocular hypotensive therapy; a normal open angle on gonioscopy; and the absence of any other ocular or systemic diseases including any brain disease that could affect the optic nerve head and/or VF. Optic disc appearance was assessed using direct ophthalmoscopy, stereoscopic observation employing a biomicroscope and appropriate lenses, and fundus photography.

IOP was measured during routine clinic visits. The spherical equivalent (SE) was determined preoperatively using an auto ref/keratometer (ARK-1; Nidek Co., Ltd.); an SE < − 6 diopters indicated high myopic eyes and an SE ≥ − 6 indicated non-high myopic eyes, based on the criteria of the International Myopia Institute [25].

**Fig. 1:**
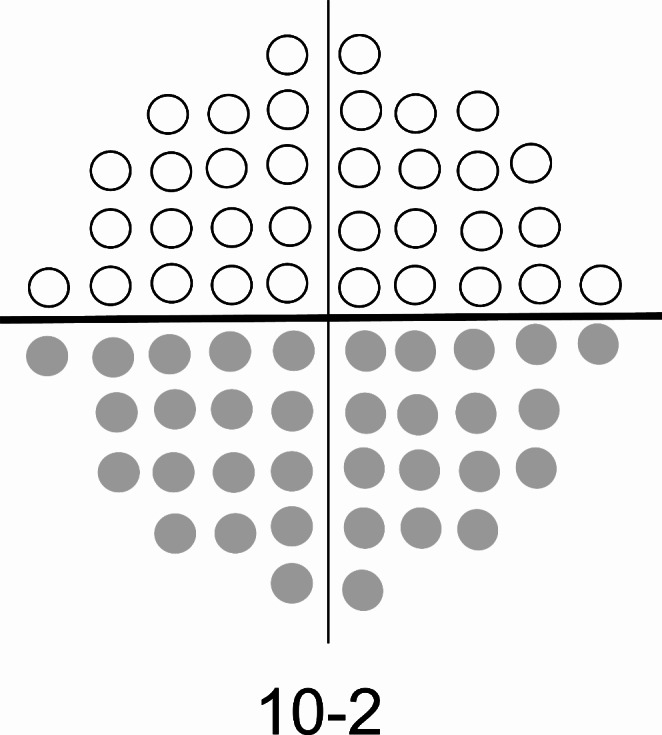
Upper and lower hemifields of the central visual field. The visual field 10-2 measurement points (68 points) were divided into upper (white circle) and lower (gray circle) hemifields by a horizontal line, with 34 measurement points included in each hemifield

Linear regression analysis was used to determine the mean deviation (MD) slope (dB/year) for the 30-2 and 10-2 VF. The total deviation (TD) values for the 10-2 VF superior and inferior hemifields (the 10-2 VF is divided into upper and lower hemifields by a horizontal line in Fig. 1) were subjected to regression analyses. Image analysis employed JGSTK Disc Analysis software (Topcon) [26, 27]. The vertical cup-to-disc ratio (v-C/D) and the PPA/disc area ratio were calculated using baseline stereo disc/peripapillary retinal and wide-angle photographs. Optic disc ovality was defined as the ratio between the longest and shortest orthogonal diameters. The optic disc torsion was the angle between the long disc (LD) and the vertical meridian (a line drawn vertically [i.e., at 90°] to a reference line) connecting the fovea and the center of the optic disc.

The surgical procedure was as follows [28]. After the instillation of a local anesthetic into the sub-Tenon space, a 5–6 mm long fornix-based conjunctival incision was created along the temporal limbus. Cauterization was then performed, and a single rectangular scleral flap (3 × 3 mm) was raised. The area was immersed in 0.05% MMC solution for 1.5 min, followed by washout with 100 mL balanced salt solution. A sclerocorneal block (0.5 mm × 2 mm in the vertical and horizontal dimensions, respectively) was then excised, and peripheral iridectomy was performed. Four 10-0 nylon sutures (MANI) were used to secure the scleral flap with adjustment of the aqueous humor flow as needed. A wing conjunctival suture was then placed. When the procedure was combined with cataract surgery, a clear corneal incision was created in the superior quadrant. The anterior chamber was filled with viscoelastic material (Viscoat 0.5 Ophthalmic Viscoelastic Substance [Alcon] with 1% Healon Ophthalmic Viscoelastic Substance [AMO]). Postoperative treatment after simple trabeculectomy involved four applications of 0.1% betamethasone and moxifloxacin ophthalmic solution. For patients who underwent combined surgery, a diclofenac sodium ophthalmic solution was added. Either laser suture lysis or bleb needling was performed at the surgeon’s discretion to enhance aqueous humor flow whenever the IOP remained elevated after surgery.

### Statistical analysis

**Fig. 2:**
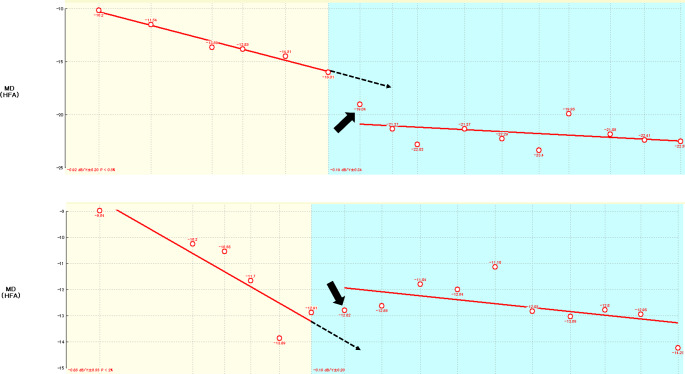
Conceptual diagram of postoperative visual field outcomes according to the rate of preoperative deterioration. The upper panel illustrates a scenario where the initial postoperative visual field (VF) is poorer than the preoperative deterioration rate predicted. Conversely, the lower panel depicts a situation where the VF is better than predicted by the preoperative deterioration rate. The dashed lines are the preoperative VF deterioration rates (regression lines), and the black arrows denote the postoperative VF test results. The horizontal axis represents the timeline (months), and the vertical axis represents the mean deviation (MD). A steeper regression line indicates a faster rate of deterioration. The background colors indicate the preoperative (yellow) and postoperative (blue) states. This figure was created using plots from BeeFiles for HFA provided by Beeline (Tokyo, Japan)

One of the main purposes of this study was to compare the rate of VF deterioration (the MD or TD slope) before and after surgery in high myopic and non-high myopic eyes. The Wilcoxon signed-rank test was used to compare the preoperative and postoperative values. After surgery, the MD of the VF may deteriorate at the same rate as preoperatively or, conversely, the MD may be better than predicted by the preoperative deterioration rate, as revealed by the postoperative MD values according to the postoperative time calculated using linear regression. Figure 2 provides schematics illustrating cases in which deterioration continued unabated and cases in which it slowed. The paired t-test was used to compare the postoperative 10-2 VF slope with versus without inclusion of the data from the first postoperative VF 10-2 tests.

R (version 3.4.3; R Foundation for Statistical Computing) and SPSS (version 23.0 for Windows; IBM) were used for all statistical analyses. A two-sided P-value < 0.05 was considered statistically significant.

## Results

Except for the PPA/disc ratio, no significant difference was observed between patients with high myopic eyes (–8.96 diopters) and non-high myopic eyes (–1.55 diopters) (Table 1). The preoperative follow-up period was 6.25 ± 1.00 years, and the postoperative follow-up period was 6.00 ± 0.61 years, for all subjects. After trabeculectomy, IOP significantly decreased from 14.1 to 9.0 mmHg (*P* ≤ 0.01) in the high myopia group and from 13.4 to 9.5 mmHg (*P* ≤ 0.01) in the non-high myopia group. The postoperative 10-2 VF deterioration rate slowed in both groups (Table 2). The first postoperative VF test was performed an average of 4.5 months after surgery; in 16 of the 28 eyes, VF was worse than the preoperative deterioration rate predicted. The postoperative deterioration rates of the two groups did not differ significantly according to whether the first postoperative VF test data were included or omitted (*P* ≥ 0.67) (Table 3).

**Table 1:** Demographic and clinical characteristics of the study patients

Characteristics	Myopic group	Non-myopic group	*P*-value
Age (years)	55.3 ± 8.2 [34–65]	59.6 ± 11.5 [28–74]	0.261
gender	Male = 4 Female = 8	Male = 2 Female = 14	0.354
Refraction (Diopter)*	-8.96 ± 2.50 [-14.5 – -6.0]	-1.55 ± 2.35 [-5.0 – +2.0]	< 0.0001
Preoperative IOP (mmHg)	13.6 ± 1.68 [10.9–15.4]	14.1 ± 1.82 [11.7–17.1]	0.450
MD (30-2) (dB)	-10.2 ± 7.57 [-23.3 – -2.3]	-9.54 ± 8.43 [-25.9–0.7]	0.827
MD (10-2) (dB)	-8.90 ± 5.26 [-19.3 – -2.6]	-11.2 ± 8.36 [-23.4 – -0.9]	0.373
PSD (30-2) (dB)	10.2 ± 5.01 [2.2–17.5]	9.85 ± 5.43 [1.7–16.0]	0.862
PSD (10-2) (dB)	9.66 ± 4.11 [1.0–14.5]	9.85 ± 5.22 [1.9–16.0]	0.913
Vertical Cup/Disc ratio	0.91 ± 0.03	0.88 ± 0.03	0.099
PPA/Disc ratio	1.01 ± 0.64	0.46 ± 0.34	0.017
Optic disc ovality	1.26 ± 0.23	1.19 ± 0.13	0.342
Optic disc rotation (degree)	-10.2 ± 31.7	-13.3 ± 39.2	0.821
Preoperative follow-up period (years)	5.92 ± 0.77 [5.1–7.3]	6.51 ± 1.14 [5.0–8.5]	0.119
Postoperative follow-up period (years)	6.03 ± 0.65 [5.0–7.3]	5.97 ± 0.61 [5.2–7.4]	0.783

Data are shown as the mean ± standard deviation

*IOP* intraocular pressure,* MD* mean deviation,* PSD* pattern standard deviation,* dB* decibels,* PPA* parapapillary atrophy

* at the time of the first visit

**Table 2:** Comparison of pre-and postoperative visual field deterioration rate in myopic and non-myopic eyes

	Myopic group			Non-myopic group		
	Pre-op.	Post-op.	P-Value	Pre-op.	Post-op.	*P*-Value
10-2 MD	-1.31 ± 0.24	-0.55 ± 0.23	0.01	-0.80 ± 0.18	-0.30 ± 0.16	0.03
10-2 Superior sector	-1.46 ± 0.30	-0.49 ± 0.18	0.005	-1.04 ± 0.24	-0.23 ± 0.097	0.02
10-2 Inferior sector	-1.31 ± 0.25	-0.61 ± 0.30	0.03	-0.53 ± 0.17	-0.42 ± 0.27	0.63
30-2 MD	-0.56 ± 0.087	-0.03 ± 0.11	0.02	-0.49 ± 0.07	-0.57 ± 0.27	0.60

Data are shown as the mean ± standard error (dB/year)

*dB* decibel,* Pre-op.* pre operation,* Post-op.* post operation,* MD* mean deviation

**Table 3:** Comparison of the postoperative rate of visual field deterioration including/excluding the first visual field test

	Myopic group			Non-myopic group		
	With the first VF	Without the first VF	P-Value	With the first VF	Without the first VF	*P*-Value
10-2 MD	-0.55 ± 0.23	-0.51 ± 0.24	0.73	-0.31 ± 0.16	-0.54 ± 0.89	0.67

Data are shown as the mean ± standard error (dB/year)

*dB* decibel,* VF* visual field,* MD* mean deviation

## Discussion

The findings of this exploratory study show that trabeculectomy effectively reduced the IOP in patients with NTG regardless of refractive status, slowing the rate of deterioration of the central VF. The postoperative central VF appeared to be worse than predicted in some patients. However, this suggests that the initial postoperative VF test may not affect the evaluation of overall progression in both myopic and non-myopic patients.

In a retrospective analysis of Japanese patients, trabeculectomy with MMC was performed on myopic eyes with glaucoma (19 eyes; mean follow-up period, 4.6 years) [29]. The success rates at 3 years did not differ between myopic and non-myopic eyes. However, the primary endpoint of the study was IOP management; the VF deterioration rate was not discussed. Another report examining the efficacy of trabeculectomy for myopic eyes with glaucoma (63 eyes) evaluated changes in the VF and retinal nerve fiber layer (RNFL) thickness after surgery [18]. There were no differences in either the pre- or postoperative MD slope between the myopic and non-myopic eyes, but significantly greater deterioration, especially in the central VF region, was observed in the myopic than the non-myopic eyes, which became more pronounced with increasing axial length (AXL). It should be noted that the central VF of the cited study was the average of the values of the central 12 points of the 24 − 2 VF, which differs from our definition (64 points). Several studies report further VF deterioration even after trabeculectomy [19, 20]. Additionally, there have been documented cases of central VF loss post-surgery [30–32]. Conversely, there have also been reports of improvements in optic disc and VF parameters following trabeculectomy [33, 34]. This may be attributable to a balance between the effectiveness of surgery and the natural progression of the disease; however, no clear distinction has yet emerged. Previous reports highlight poor baseline VF function as a risk factor for postoperative VF deterioration, emphasizing the need for caution during surgical management particularly in patients with advanced disease [35, 36]. When considering trabeculectomy for severe cases, it is crucial to inform patients about the high risk of postoperative central VF deterioration. Our study identified a poorer MD and a smaller PSD as potential risk factors for central VF deterioration (data not shown).

Considering real-world clinical setting and the unavailability of axial length (AXL) measurements for all participants, we used refractive values to classify myopia. This approach differs from previous studies that primarily focused on axial myopia, including eyes with refractive myopia. While myopia can be classified using refractive data or AXL, AXL may be particularly relevant in clinical settings where the risk of myopic glaucoma is assessed. Notably, the highly myopic eyes often exhibit structural abnormalities in the sclera and lamina cribrosa, rendering the optic nerve more vulnerable to damage. Previous studies have used AXL for myopia classification. However, AXL does not consistently align with elongation towards the optic disc. Moreover, eyes with longer AXLs may exhibit proportionally larger other optical components. A limitation of our study was the lack of AXL measurements, hindering a comprehensive evaluation of the association between myopia and potential structural changes. Prior studies, including meta-analyses and longitudinal studies such as the Ocular Hypertension Treatment Study [37], have used classifications based on refractive error [38–40]. In our study, to ensure accurate refraction measurements, all cases with nuclear cataracts were excluded from the analysis.

Another limitation of our study was its retrospective design. This introduced a degree of patient bias, particularly because of the small number of patients followed up before and after surgery. It is difficult to accumulate reliable data over a long period of time in actual clinical practice. Reliable evaluation of VF deterioration is possible only after a certain number of VF tests have been conducted [41], which inevitably limited the number of cases include; more cases will be required in future studies.

Also, distinguishing between retinal damage associated with myopia and NTG can be exceedingly challenging, particularly given the myriad clinical findings associated with high myopia. In one retrospective study targeting suspected myopic glaucoma patients under 50 years of age, myopia was associated with VF defects resembling those seen in glaucoma, such as a nasal step and arcuate and parafoveal scotomas [42]. Although such defects may be attributable to myopia rather than glaucoma, differentiating between the two is often challenging. Myopia is known to cause various VF abnormalities similar to those characteristic of glaucoma, but unlike glaucomatous defects, these typically do not progress over time in untreated eyes. Therefore, long-term observation is a critical aspect of glaucoma assessment in myopic eyes [43, 44]. Although examination of progression for at least 5 years before surgery does not guarantee the absence of myopic VF changes (as stated in the inclusion criteria), we believe that the likelihood thereof is significantly reduced. In other words, we have a high level of confidence that such changes are absent. Our focus was not on the presence or absence of myopic VF changes; rather, it was only on the effect of filtration surgery on postoperative VF.

Another limitation of our study was the incomplete collection of post-washout IOP data. Only patients with available post-washout IOP measurements were included in the analysis, potentially introducing bias. Future studies should implement more comprehensive washout protocols and collect data from all participants to mitigate this limitation. Finally, there is no standardized timeframe within which the first VF test should be conducted after filtering surgery. This suggests that, at least in patients with advanced NTG, it is possible that progression may not be suppressed within 6 months after surgery. In our population, the first postoperative VF test was performed an average of 4.5 months post-surgery. Although it may not be necessary to perform VF tests within the first 6 months after surgery, particularly in patients with advanced disease, the key question was whether the timing of surgical intervention was appropriate in this study; this was entirely at the surgeon’s discretion. We evaluated progression with and without the first postoperative VF test. Considering the inherent variability of VF testing, a single result is insufficient for evaluating progression. Instead, multiple tests are needed to assess changes over time. Therefore, the inclusion or exclusion of the first postoperative VF test may not accurately reflect the impact of surgery on progression. If some cases exhibiting slow progression had been included even before surgical intervention, the interpretation of the results would have been very different.

In summary, the findings of this study suggest that trabeculectomy may reduce central VF deterioration in NTG patients with or without refractive myopia. The data suggest that some patients exhibited glaucoma progression within 6 months postoperatively, but in the long term, any effect of this on evaluations of postoperative progression appeared to be minimal.
